# Full-Scale Prestress Loss Monitoring of Damaged RC Structures Using Distributed Optical Fiber Sensing Technology

**DOI:** 10.3390/s120505380

**Published:** 2012-04-27

**Authors:** Chunguang Lan, Zhi Zhou, Jinping Ou

**Affiliations:** 1 School of Civil Engineering, Harbin Institute of Technology, Harbin 150090, China; E-Mails: lcg98011210@163.com (C.L.); oujinping@dlut.edu.cn (J.O.); 2 School of Civil Engineering, Dalian University of Technology, Dalian 116024, China

**Keywords:** optical fiber sensor, Brillouin optical time domain analysis (BOTDA), smart steel strand, full-scale prestress loss, prestressed structure with damages

## Abstract

For the safety of prestressed structures, prestress loss is a critical issue that will increase with structural damage, so it is necessary to investigate prestress loss of prestressed structures under different damage scenarios. Unfortunately, to date, no qualified techniques are available due to difficulty for sensors to survive in harsh construction environments of long service life and large span. In this paper, a novel smart steel strand based on the Brillouin optical time domain analysis (BOTDA) sensing technique was designed and manufactured, and then series of tests were used to characterize properties of the smart steel strands. Based on prestress loss principle analysis of damaged structures, laboratory tests of two similar beams with different damages were used to verify the concept of full-scale prestress loss monitoring of damaged reinforced concrete (RC) beams by using the smart steel strands. The prestress losses obtained from the Brillouin sensors are compared with that from conventional sensors, which provided the evolution law of prestress losses of damaged RC beams. The monitoring results from the proposed smart strand can reveal both spatial distribution and time history of prestress losses of damaged RC beams.

## 1. Introduction

During the past decades, prestressing techniques were frequently used in the modern buildings to reduce the deadweight of structures and improve their durability and reliability. Typical application examples can be seen in the construction of bridges and nuclear reactor containments [1,2]. Considering that existing actual stress in tendons is related to the active state of prestressed concrete structures, the prestress loss is of paramount importance. In general, prestress losses are considered finished with the construction stage and are ignored during the in-service phase. With the rapid development and wide application of concrete additives, prestressed concrete structures can now be in-service before prestress losses have finished. In the service process, prestress losses increase accordingly with the damage to the prestressed concrete structures [3], so it is very important to examine the evolution law of prestress losses in damaged prestressed structures.

With the need to monitor the prestress loss under service, many approaches have been developed to implement the measurement in the structures. Ahlborn *et al.* [4] applied acoustic emissions to monitor the prestress loss under vehicle load conditions. Wu *at el.* [5] improved the accuracy of the acoustic source location techniques based on the number of sensors used. Chen *et al.* [6] and Scalea *et al.* [7] used the concept of acoustoelasticity (change in ultrasonic velocity with applied stress), coupled with the elongation effect, for the measurement of stress levels in post-tensioning rods and seven-wire strands. Maji *et al.* [8,9] obtained the stress of the strands at random time using shaped memory alloy (SMA) sensors. Wang *et al.* [10,11] developed magneto-elastic sensors to monitor the stress in a multi-strand-cable system, then applied these sensors on the Qianjiang 4th bridge to monitor the stresses of key hanger cables and post-tensioned cables. Kim *et al.* [12] presented a vibration-based method to simultaneously predict prestress loss and flexural cracking in PSC girder ridges. Barr *et al.* [13,14] monitored the behavior of five prestressed concrete girders made with high-performance concrete using vibrating-wire strain guages over a period of approximately three years. Because of the installation difficulties, it is hard to monitor the prestress losses of real structures in various applications.

In recent years, optical fiber (OF) sensors have been increasingly applied to monitor prestress losses due to their distinguishing advantages of corrosion resistance, high accuracy, electromagnetic interference resistance, capability of (quasi-)distributed and absolute measurement [15–19]. Maaskant *et al.* [20] fixed bare fiber Bragg grating (FBG) sensors on three kinds of tendons (steel strands, carbon fiber reinforced plastics rebars, graphite rods) in six prestressed concrete beams of the Beddington highway bridge to monitor time-dependent stress of prestressed tendons. Inaudi *et al.* [21] employed long gauge sensors to evaluate the curvature variations and calculate the horizontal and vertical displacements by double integration of the curvatures. Jiang *et al.* [22,23] developed a force-testing ring with temperature compensation based on optical fiber Bragg gratings (FBG) and fixed the proposed force ring on the Wuhan Yangluo Yangtze river bridge to monitor the stress of the cables. Lin *et al.* [24] applied fiber Bragg grating (FBG) sensors to investigate the behaviour of prestressed concrete beams under sustained loading. Shi *et al.* [25] bonded fiber optic Brillouin-OTDR distributed strain sensors to measure the stress of four post-tensioning cables [one steel strand and three Aramid fiber reinforced plastic (AFRP) cables]. Idriss *et al.* [26] installed long-guage (2 m total length) optical fiber deformation sensors into girders of the Rio Puerco and I-10 Bridge over University in Las Cruces in order to evaluate *in-situ* material properties, prestress loss and cambers in the girders. Xuan *et al.* [27] presented an optical fiber sensor to quantitatively evaluate the prestress losses in steel-strand reinforced structures, and employed 14 optic fiber sensors on the steel-strands through the pre-designed windows on a sewage treating tank.

Despite many efforts, the available optical sensors for prestress loss monitoring have a common application problem, which is related to the accessibility of installation and ruggedness during application, especially their long-term survivability in harsh environments. Zhou *et al.* [28] integrated FBG sensors into fiber-reinforced polymer (FRP) rebar to improve their ruggedness and they further (Zhou *et al.*; Deng *et al.* [29–32]) applied the smart FRP rebar on cables and steel strands to monitor the long-term stress of steel strands in service. In this paper, a novel smart steel strand based on Brillouin optical time domain analysis (BOTDA) technique was designed and manufactured, and series of tests were used to characterize the properties of the proposed sensor. Laboratory tests of two similar beams with different damages were used to verify the concept of monitoring full-scale prestress loss of damaged beams using the smart steel strands. The prestress losses have been measured by the proposed smart steel strand and the monitored results from the smart steel strand were compared with those from conventional sensors to examine the prestress loss evolution of damaged beams.

## 2. Prestress Loss Principle of Damaged RC Structure

**Figure 1.**
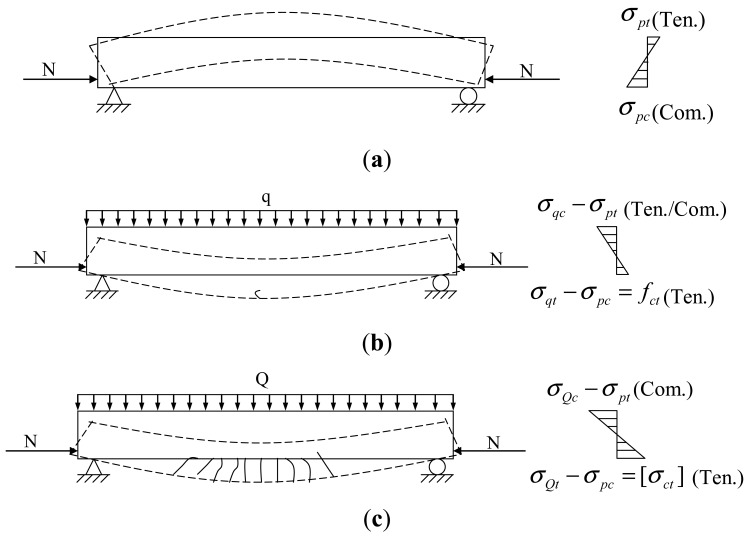
Stress analysis of RC simple supported beam. (**a**) Prestress construction; (**b**) Initial cracking; (**c**) Normal service limit.

From stretching prestressed tendons to ultimate bearing failure, there are three working states of prestressed structure: prestress construction, serviceability limit and bearing capacity limit. Damaged PC structures work in the serviceability limit state. According to the degree of damage, there are two states of initial cracking and normal service limit. Figure 1 shows the stress analysis of a simple supported PC beam in the initial cracking and normal service limit states.

### 2.1. Initial Cracking Stage

In the prestessed beam there are upper surface tensile stresses (*ó_pt_*) and lower surface compressive stresses (*ó_pt_*) caused by prestress forces (shown in Figure 1(a)). At the in-service stage, the beam stress caused by service loads is inversely related with that due to prestress force. The tensile stress (*ó_qt_*) increases with service load. During the initial stage, e.g., the value of tensile tress (*ó_qt_*) is equivalent to that of compressive stress (*ó_pt_*), the stress of lower surface in prestressed beam is zero, and then it becomes tensile stress. When the tensile stress of the lower surface reaches the ultimate tensile strength of concrete (*f_ct_*), the first crack appears, defined as the initial cracking stage (shown in Figure 1(b)). In such avstate, the beam has cracks (one or two) with a width of approximately 0.05 mm. Because of the elastic mechanical properties of beams, the cracks will be completely closed and the stress will recover to the no-load state, so the prestress losses can be neglected in the initial cracking state.

### 2.2. Normal Service Limit State

With increasing service load a series of vertical cracks will appear on the surface of the beam, while the width of existing cracks increases. When the crack width reaches the maximum allowable value (the tensile stress of the lower surface is equivalent to the allowed stress (*ó_ct_*) corresponding to crack width limits), the prestressed concrete beam is under the limit state, defined as the normal service limit stage (shown as Figure 1(c)). Considering that the prestress tendons are the main load-bearing components and brittle materials with high elastic stress segments, the prestressed beam displays elastic mechanical behavior in the normal service limit stage, so when the service load is unloaded, most of the existing cracks will be closed, but because of concrete chipping off nearby the cracks, the prestress loss that will result from that the beam becomes shorter. The prestress losses are non-uniformly distributed along the beam and increase as more cracks occur.

## 3. Distributed Optical Fiber Brillouin Smart Strand

### 3.1. Sensing Principle of the Smart Steel Strand

**Figure 2.**
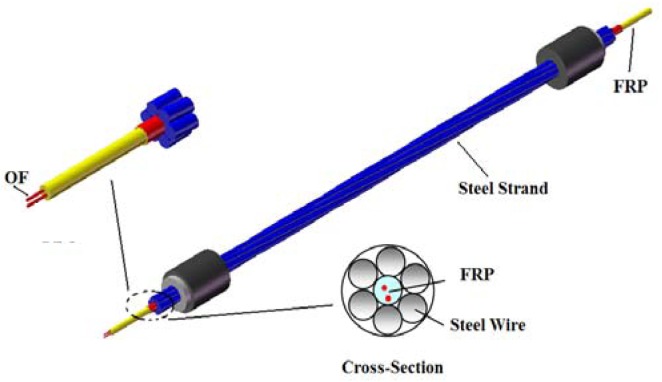
Sketch of smart steel strands structure.

Optical fiber sensors have been developed for a number of years and many optical fiber-based sensing techniques have been established for structural health monitoring because of their advantages such as immunity to electrical noise, long-term measurement stability and resistance to corrosion. In this study, the smart steel strand is a steel strand with sensing capability for structural condition assessment using BOTDA sensors. The optical fiber (OF) sensor was embedded into a fiber reinforced plastic (FRP) rebar of 5 mm in diameter, named as FRP-OF rebar, to enable its sensing capability. The smart FRP rebar was covered with copper foils and then embedded in the middle of a steel strand, which consisted of six common steel wires around (shown in Figure 2). As the FRP rebar deforms together with the remaining six steel wires, the deformation of the steel strand can be directly measured by the optical fiber sensors embedded in the rebar.

**Figure 3.**
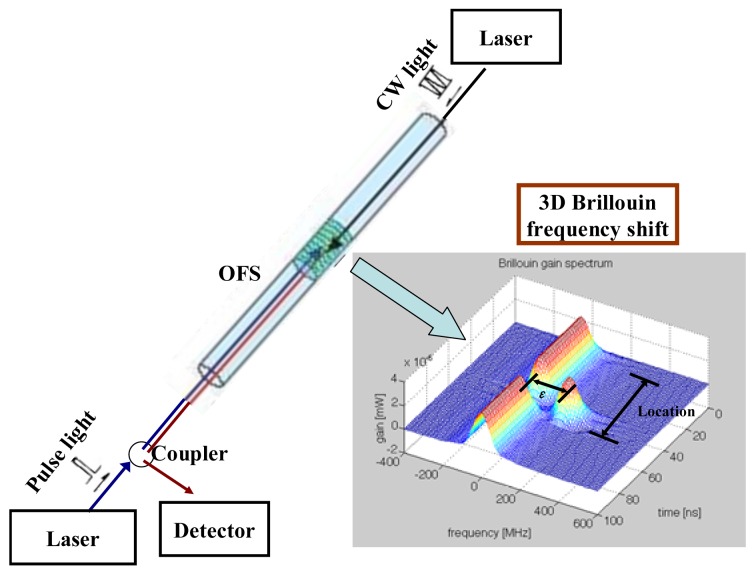
Principle diagram of fiber optic BOTDA technology.

Instead of a normal strand, the smart steel strand was anchored in a prestressed concrete structure. Then the prestress loss of the strand can be monitored by optical fiber sensors in the smart steel strand using BOTDA technology. The principle diagram of fiber optic BOTDA technology is shown as Figure 3.

When an optical pulse is launched into the optical fiber in the smart steel strand, some backscattered signals return to the input end. There are three main types of scattering, and Brillouin scattering is one of them. The pumping pulse light is launched at one end of the fiber and propagates in the fiber, while the continuous wave (CW) light is launched at the opposite end of the fiber and propagates in the opposite direction. When the power of optical pulse signal, which propagates along the single-mode optical fiber, is larger than the Brillouin threshold power, the backward stimulated Brillouin scattering signal is generated. Stimulated Brillouin scattering signal can be described as a parametric interaction among the incident light, the Stokes light, and an acoustic wave. The Brillouin frequency shift *ν_B_* of the backward scattering light of the propagating light in an optical fiber is given by [33] as bellow:

(1) $$v_{B}=\frac{2nv_{A}}{\lambda_{p}}$$

where *ν_A_* is the velocity of light wave propagating in vacuum, *n* is the effective refractive index of the optical sensing fiber and *λ_p_* is the wavelength of the input optical pulse.

When a strain (*ε*) is applied or the temperature (*T*) is varied on the fiber, this Brillouin frequency shift *v_B_* changes linearly with the applied strain and temperature differences [34,35]. It is expressed as:

(2) $$v_{B}(ɛ,T)=v_{B0}+C_{ɛ}ɛ+C_{T}T$$

And for a temperature compensating sensor without applied strains, its frequency shift *v_BT_* (0, *T*) only depends on the temperature differences. Thus:

(3) $$v_{BT}(0,T)=v_{B0}+C_{T}T$$

Then the applied strains on the optical sensing fiber yields to:

(4) $$ɛ=\frac{\Delta v_{B}(ɛ)}{C_{ɛ}}=\frac{v_{B}(ɛ,T)-v_{BT}(0,T)}{C_{ɛ}}$$

where Δ*v_B_(ε)* is Brillouin frequency shift with strain; *v_B_*(0,*T*) is Brillouin frequency shift without strain and about 11 GHz; *C_ε_* is the sensing coefficient of strain and about 0.5 GHz/% (strain) at the wavelength k = 1.55 μm. According to Hooke's law, the stress (*ó*) of the stand can be shown as:

(5) $$\sigma =E\Delta v_{B}/C_{ɛ}$$

where *E* is the equivalent Young's module of the smart strand.

Finally, the prestress loss (*ó_l_*) of the strand can be expressed as:

(6) $$v(ɛ,T)=v(0,0)+C_{ɛ}ɛ+C_{T}T$$

where *ó_con_* is the controlling stress of the strand.

### 3.2. Manufacture of the Smart Steel Strand

**Figure 4.**
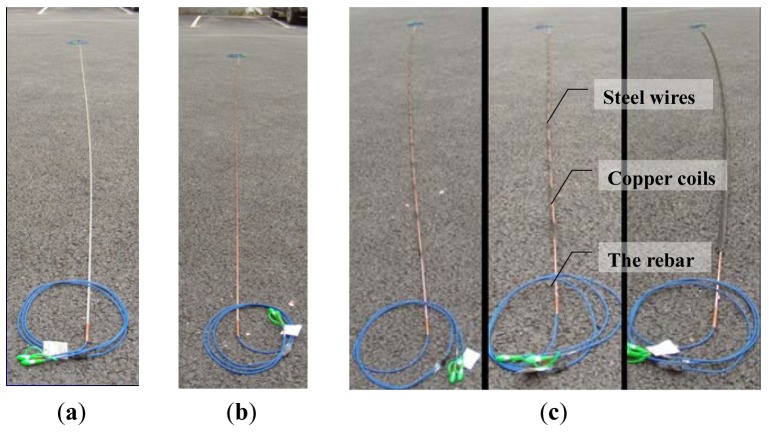
Photos of manufacturing the smart steel strand. (**a**) The rebar; (**b**) Winded the coil; and (**c**) Assemble the smart strand.

Figure 4 shows the manufacturing process of the proposed smart steel strands. To protect the FRP-OF sensor and increase the bonding between the smart rebar and the steel bars, copper coils were fixed on the FRP-OF rebar using No. 502 glue. For each cross section of the rebar, two or three layers of copper coils were clipped. The common steel strands were then cut to a certain length according to the design. The six steel wires are separated clockwise or anticlockwise, and the sequences of the wires are remembered. The smart steel strand was finally completed by winding the six steel strands with the FRP-OF rebar in turn with the contrary sequence.

### 3.3. Calibration of the Smart Steel Strand

**Figure 5.**
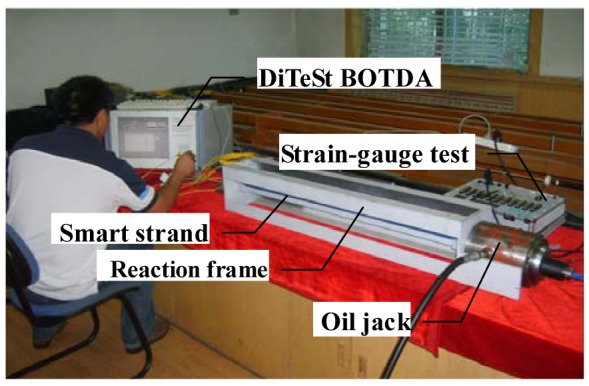
Setup of calibration tests.

To characterize the sensing properties of the proposed smart steel strand, one smart steel strand was manufactured following the procedure described in the last section and tested in the laboratory for calibration. Figure 5 shows the experimental setup for the calibration test. The test specimen had a length of 3 m and was fixed on a reaction frame. The hydraulic jack used in calibration test has a limited loading range, and the purpose of the calibration test is to investigate the sensing performance of the smart steel strand, so the load used was 30 kN, divided into nine loading steps of 3.33 kN for each step. Three loading cycles were repeated. The strain of strand (measured by a DiTeSt STA2000 apparatus produced by Omnisens, Switzerland) was recorded.

**Figure 6.**
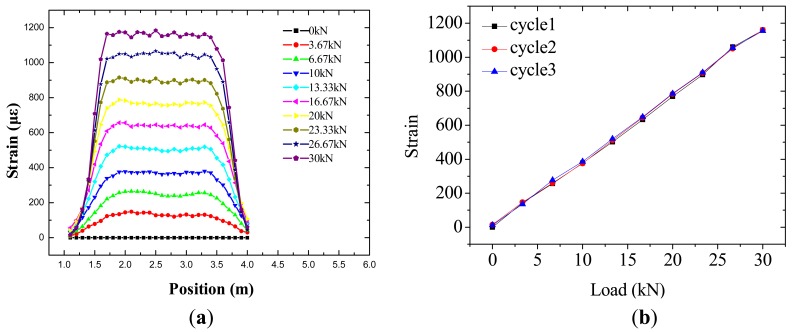
Results of the calibration test. (**a**) Strain distribution; (**b**) Relation between loads and strain at the position 2.5 m.

The smart steel strands are sufficient and easy to install. Figure 6(a,b) shows the calibration results of the smart steel strand obtained from the BOTDA sensors. The full-scale strain distribution of the strands can be obtained by the BOTDA sensors. The BOTDA sensor in the smart steel strands has a good linearity, with linear coefficients of 99.948%. The load sensitivity of the BOTDA sensor yields 38.824 με/kN. The resolution along the length of the fiber was 10 cm. Thus, the proposed FRP-OF smart steel strands gives promising results and could be applied for further structural property investigation of the steel strands.

For static performance indexes not only showing sensing properties of the sensors, but also affecting dynamic indexes, it is particularly important to analyze the static characteristics. Main static indexes such as hysteresis, linearity, repeatability and overall accuracy *etc.* are used to describe the application of a sensor under actual conditions and evaluate the merits of sensors. According to the Chinese National Standard “Methods for calculating the main static performance specification of transducers” (GB/T 18459-2001), the main static performance indexes of the smart strand was computed using the calibration test data. Table 1 shows the main static performance indexes of the smart steel strand, which are linearity of 3.9% FS (full-scale), hysteresis of 1.3% FS, repeatability of 1.7% FS and overall accuracy of 3.3% FS.

## 4. Experimental Work

### 4.1. Experimental Procedure

**Figure 7.**
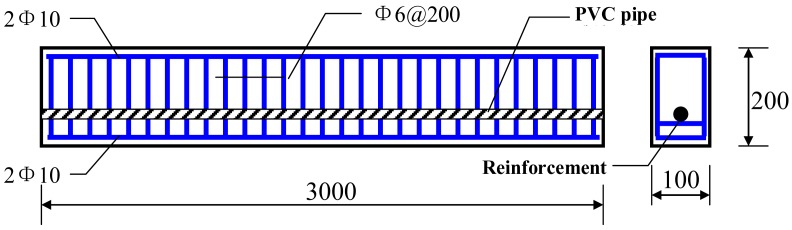
Reinforcement of the beam (unit: mm).

Two unbounded prestessed concrete beams were designed and cast with the same dimensions and materials. The concrete beams (compressive strength is 26.8 MPa) tested in this series of experiments had a span of 3 m and a cross-section of 100 mm × 200 mm. One proposed smart steel strand was implemented as prestressed reinforcement in the concrete beam. The smart strand had a diameter of 15.12 mm and a standard strength (*f_ptk_*) of 1,660 MPa. Four common steel reinforcements (yielding strength is 310 MPa) with a diameter of 10 mm were distributed in the tension region and compression area (shown in Figure 7). Steel plates and spiral reinforcements were embedded in the tension and anchoring region of the beam to eliminate the stress concentration. The thickness of the steel plate was 10 mm, and the spiral reinforcement was 4 mm in diameter, 50 mm in spiral inner distance. The concrete beams were cured for 28 days in room temperature after casting.

**Figure 8.**
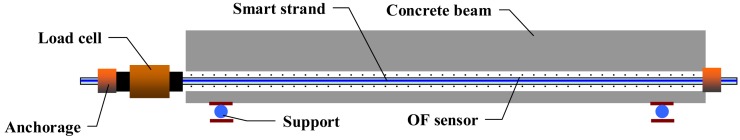
Scheme of sensor location in the prestressed concrete beam.

In the fiber reinforced plastic (FRP) optical fiber (OF) rebar, one optical fiber sensor had been applied in the full-length of the beam. To ensure the stability of the sensor, there were no weaknesses (solder joints, *etc.*) on the optical fiber sensors. To validate the smart steel strand, a load cell was installed at the tension end for comparison, as shown in Figure 8.

**Figure 9.**
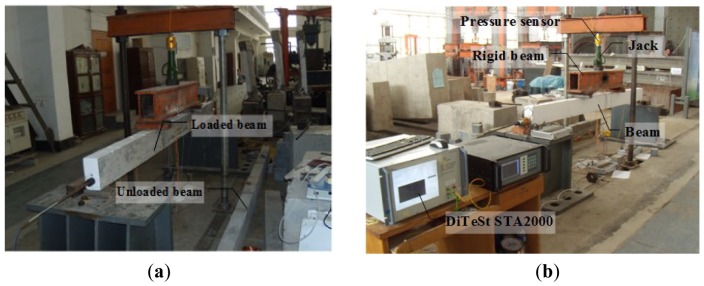
Experimental setups of prestress losses monitoring test. (**a**) Two experimental beams; and (**b**) Experimental equipment.

Figure 9 shows the test setup. The smart strand was tensioned by a hydraulic jack and anchored by a single-hole anchorage. The control stress applied in this test was 0.70*f_ptk_*, resulting in a max tension load of 160 kN. After 20 days, two concentrated loads provided by the hydraulic jack were applied to a location 500 mm from the center of the loaded beam. At same time, the other beam (unloaded beam) did not bear any loads. The pressure sensor was implemented for the loading control.

Here, two damage conditions (initial cracking and normal service limit state) of prestressed beam were investigated. To analyze time-dependent prestress loss of the concrete beam at the stage of first crack occurrence, all sensors started to record data at the stage of crack initiation and unloading to zero. Data were also recorded by the load cell and the smart steel strand as the limiting crack width then unloading to zero for analyzing time-dependent prestress loss of the concrete beam in the normal service limit state.

### 4.2. Experimental Results and Discussions

**Figure 10.**
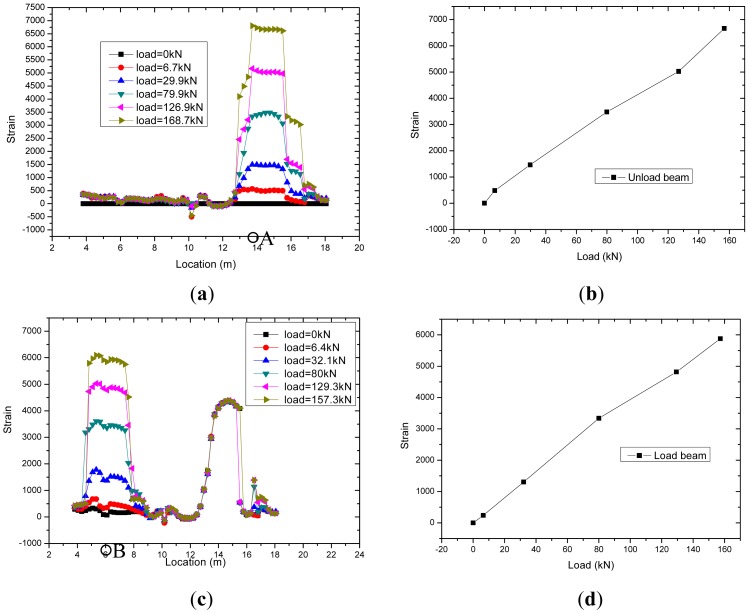
Strain results during the tensioning operation monitored by BOTDA sensors in the smart strand. (**a**) Results of unloaded beam; (**b**) Results of point A; (**c**) Results of loaded beam; (**d**) Results of point B.

Figure 10 shows the strain results during the tensioning operation as monitored by the BOTDA sensors. In order to save channel numbers of Brillouin demodulator and test time, the optical fiber sensors of loaded and unloaded beams were connected in the data acquisition process. From Figure 10(a,c), the test length of strands in the loaded beam (*x* axis 5–8 m) and unloaded beam (*x* axis 13–16 m) can be clearly distinguished. From Figure 10(b,d), the strain measured by the optical fiber sensors in the two beams increased linearly with increasing loads, and the correlation coefficient is 99.8%. The sensitivity coefficient of optical fiber sensors in two beams is approximate, and the sensitivity coefficients in loaded beam and unloaded beam are 40.6 με/kN and 37.6 με/kN, respectively.

**Figure 11.**
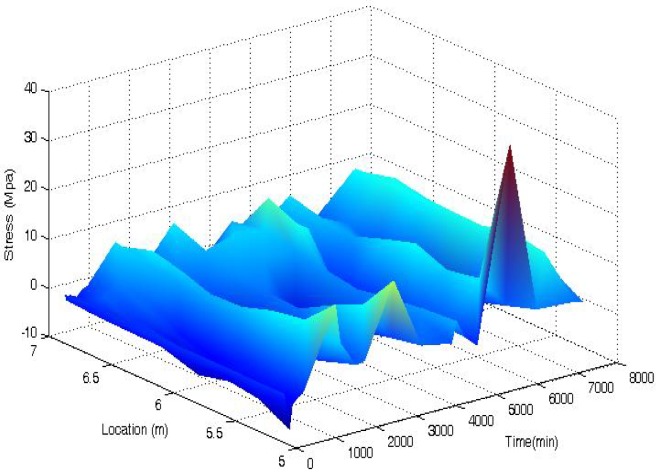
Time-dependent prestress losses of prestressed beam at the initial cracking stage measured by the BOTDA sensors in the smart strand.

A Figure 11 shows the 3-D time-dependent prestress losses of the beam measured by the BOTDA sensors in the smart steel strand at the stage of first crack occurrence. The full-scale strain (location 5–7 m) distribution of the strand can be obtained by the BOTDA sensors. The minor damages of the beam do not change the prestress loss significantly, except for a single catastrophe point (the peak near the location of 6 m and at the time of 7,000 min) due to the boundary effect of the BOTDA testing apparatus.

**Figure 12.**
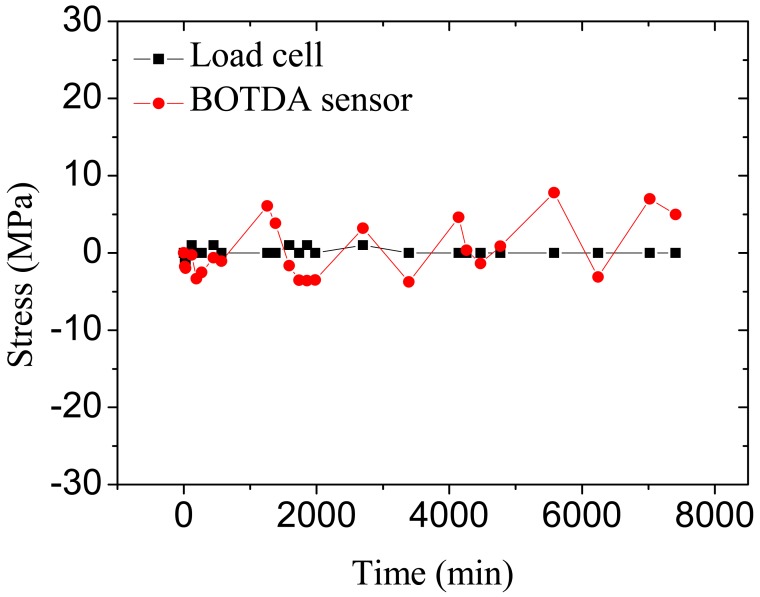
Prestress losses of the prestressed beam at the initial cracking stage measured by the BOTDA sensor and load cell.

Figure 12 shows the time-dependent prestress loss results at the mid-span cross section of the beam measured by the BOTDA sensors and the load cell at the stage of the first crack occurrence. It shows that the stress measured by the BOTDA sensor agrees well with that determined by the load cell. With a lower resolution, the results monitored by BOTDA sensor fluctuate along that measured from the load cell. The time-dependent prestress loss of the beam with small damages (cracks) fluctuates slightly with time, indicating that minor damages are negligible for prestress loss.

**Figure 13.**
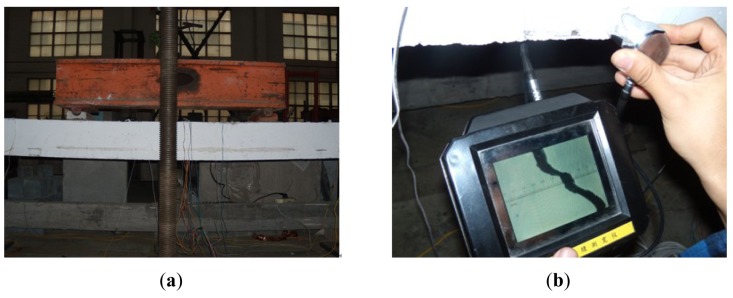
Photos of cracks distribution and measurement. (**a**) Crack distribution; and (**b**) Measuring crack width.

Figure 13 shows photos of the cracks distribution and the measurement of crack width. When the load was 10 kN, the first vertical crack appeared on the left loading position of the prestressed concrete beam. With the increasing loads, the width and number of vertical cracks increased, then the diagonal cracks appeared on two loading positions, one after another. When the load was 50 kN, the maximum crack width of lower edge of prestressed concrete beam reached 0.2 mm.

**Figure 14.**
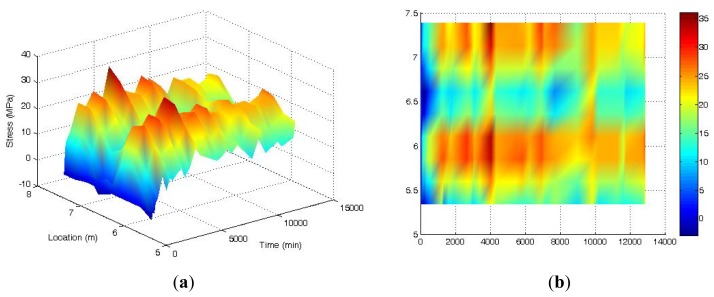
Prestress losses of prestressed beam at normal service limit state measured by BOTDA sensor in the smart strand. (**a**) Three-dimension graph; and (**b**) Stress nephogram.

Figure 14 shows the 3-D time-dependent prestress loss of the loaded beam measured by the BOTDA sensors in the smart steel strand at the normal service limit state. The full-scale strain (location 5.5–7.5 m) distribution of strand can be obtained by the BOTDA sensors. The change of prestress loss is highly related to the distribution of cracks. The prestress losses are not uniformly distributed along the beam, with the locations of 6 m and 7 m being larger than other locations. This is because the loads were directly placed at these locations and more cracks were observed in this region. The prestress losses then are indicated to be increased as more cracks occur.

**Figure 15.**
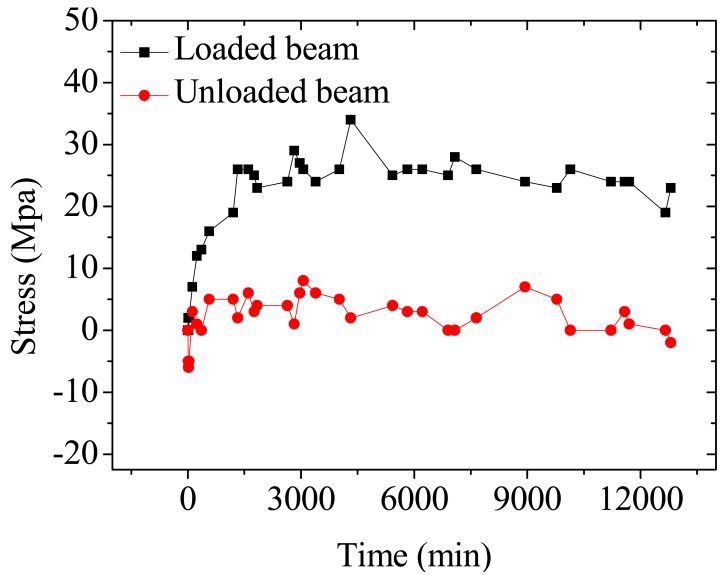
Time-dependent losses of prestressed beam at normal service limit state monitored by BOTDA sensors in two beams.

Figure 15 shows the test results of time-dependent prestress loss at the mid-span cross section of two beams measured by the BOTDA sensors after the occurrence of the maximum allowable crack width (0.2 mm). The prestress losses of the loaded beam monitored by the BOTDA sensors increases with time, during the initial stage, e.g., in the first day, a higher prestress loss rate is observed, and that of the unloaded beam do not change with time.

## 5. Conclusions

In this paper, based on optical fiber Brillouin sensing technology, a novel smart steel strand was designed and manufactured to monitor full-scale prestress loss of prestressed structures. Calibration tests were used to characterize the sensing properties of the proposed sensor. Finally, laboratory tests of two similar beams with different damages were used to verify the concept of full-scale prestress loss monitoring of damaged beams using the smart steel strands. The prestress loss was obtained from the BOTDA sensors. The results show that the proposed smart steel strands are sufficiently rugged and easy to install using normal equipment, the smart steel strand has a sensitivity coefficient of 43.98 με/kN, linearity of 3.9% FS, hysteresis of 1.3% FS, repeatability of 1.7% FS and overall accuracy of 3.3% FS, and full-scale prestress loss of damaged structures can be obtained by distributed optical fiber smart steel strands. The time-dependent prestress loss of beams at the stage of the first crack occurrence doesn't change significantly with time. The prestress losses of beams at the stage of maximum allowable crack width increased with time. During the initial stage, e.g., on the first day, a higher prestress loss rate is observed and the rate of prestress loss reduces gradually then after.

## Figures and Tables

**Table 1.** Static performance indexes of the smart steel strand.

**Static performance indexes**	**Calculation method**	**Calculation result**
Linearity	$\xi_{L}=\frac{|{\bar{y}}_{i}-y_{i}{|}_{\max}}{y_{\max}-y_{\min}}\times 100\%$	3.9%
Hysteresis	$\xi_{H}=\frac{|\Delta {\bar{y}}_{i}{|}_{\max}}{Y_{\max}-Y_{\min}}\times 100\%$	1.3%
Repeatability	$\xi_{R}=\frac{\left(2\sim 3\right)S}{y_{\max}-y_{\min}}\times 100\%$	1.7%
Overall accuracy	$A=\sqrt{{\xi_{H}}^{2}+{\xi_{L}}^{2}+{\xi_{R}}^{2}}$	3.3%
